# Surgical treatment of a presacral schwannoma: A case report

**DOI:** 10.1016/j.amsu.2022.104609

**Published:** 2022-09-15

**Authors:** Magherbi Houcine, Ouadi Yacine, Hammami Mahdi, Zehani Alia, Boukriba Seif, Zaraa Mourad, Fterich Fadhel Samir, Montasser Kacem

**Affiliations:** a Department of Surgery A La Rabta Hospital, Tunis, Tunisia; b Faculty of Medicine of Tunis, Tunis El Manar University, Tunis, Tunisia; c Department of Radiology La Rabta Hospital, Tunis, Tunisia; d Department of Orthopedic Surgery, CTGB Hospital, Tunis, Tunisia; e Department of Pathology, La Rabta Hospital, Tunis, Tunisia

**Keywords:** Case report, Schwannoma, Neurosurgery, General surgery

## Abstract

**Introduction and importance:**

Schwannomas are rare benign tumors that develop from Schwann cells that represent 0.3 to 0.4 cases per 100,000 persons per year. We report a case of pre-sacral schwannoma, a rare tumor, especially in the pelvic area. This case comes to help further teams in their management as its scarcity made any attempt to make proper recommendations obsolete.

**Case presentation:**

a 53-year-old otherwise healthy woman who presented with a 2-year history of right-sided sciatica. The radiologic characteristics of the mass suggested presacral schwannoma type III of the Klimo classification as the most possible diagnosis.

Therefore, surgical resection was decided, and an anterior approach was chosen. By laparotomy a 6cm retroperitoneal encapsulated mass with no invasion of the adjacent organs. We performed a digital enucleation of the tumor through a capsulotomy.

Pathology confirmed the diagnosis of schwannoma. There were no features of malignancy. The post-operative period was uneventful. A follow-up examination at 6 months showed no signs of numbness or weakness in the right leg. The previously described pain totally regressed.

**Discussion:**

Although schwannoma is a benign lesion, it may become malignant, especially when associated with neurofibromatosis making its surgical removal primordial. Its pelvic location may make its diagnosis delayed due to non-specific symptoms mainly through compression of local organs. Its surgical management can be challenging due to large size tumors with adherence to peritoneal and retroperitoneal organs. Quality of the resection is important in the recurrence and necessity for reoperation. A multidisciplinary approach is therefore recommended to ensure optimal treatment

**Conclusion:**

Due to its rareness, there is no clear consensus in on the management of schwannomas therefore we chose to write our case in order to further enrich the literature to achieve one-day guidelines for schwannoma treatment.

## Highlights

• Schwannoma is rare.
• Symptoms are nonspecific.
• Surgery is the only permanent solution.
• Surgical approach depends on shwannoma type.
• Recurrence is possible in case of incomplete surgery.

## 1 Introduction and importance

Schwannomas are mostly benign tumors that develop from Schwann cells. They are usually found in the neck and head region, but sporadic cases of pelvic schwannomas have been reported. They are usually asymptomatic until their growth compresses adjacent organs. Due to inconsistent symptoms, they are often misdiagnosed as pelvic lesions. We report a case of pre-sacral schwannoma, a rare tumor, especially in the pelvic area. This case comes to help further teams in their management as its scarcity made any attempt to make proper recommendations obsolete.

This case report has been reported in line with the SCARE Criteria [1].

## 2 Case presentation

We report the case of a 53-year-old otherwise healthy woman who presented with a 2-year history of right-sided sciatica. The pain was greater in the supine position and slightly relieved when upright. There was no weakness or numbness. The symptoms began gradually 2 years ago with no history of trauma or injury.

**Fig. 1:**
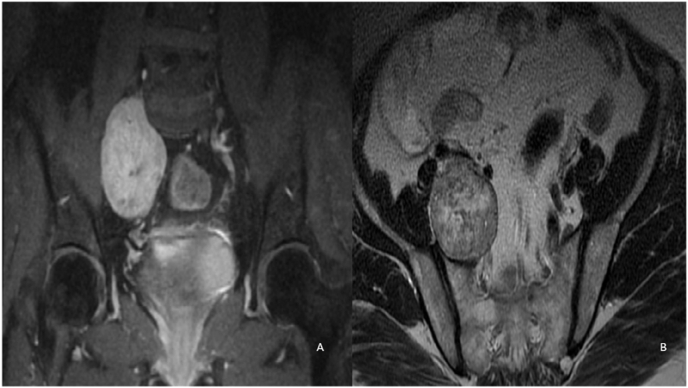
Retroperitoneal mass emerging from the right foramen of L4-L5 ovaler, well circumbscribed with smooth margins. This mass is in high heterogenous signal in T2 coronal Fatsat sequence (A) and in axial T2 sequence (B).

Initial management included an EMG that showed myelin damage to the right L5 root which indicates L5 root compression. MRI of the pelvis and lumbar-sacral spine revealed a well-defined ovoid mass in the right presacral space, adherent to the sacral anterior surface without any sign of invasion growth. The parenchyma was heterogeneous with few calcifications noted. The mass had contact with the external iliac vein, internal iliac artery and vein, and the right ureter (Fig. 1). The radiologic characteristics of the mass suggested presacral schwannoma type III of the Klimo classification as the most possible diagnosis.

Therefore, surgical resection was decided, and an anterior approach was chosen. We found it necessary to put a double J stent in the right ureter prior to the operation to prevent its injury during the pelvic dissection.

**Fig. 2:**
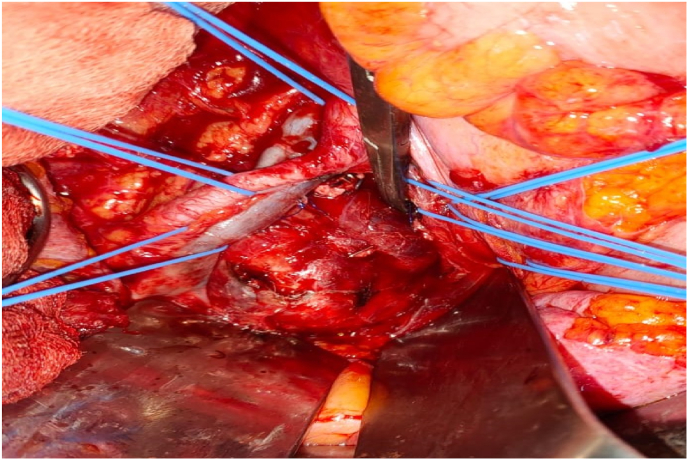
Picture showing the schwannoma after its dissection from the aorta and common iliac bifurcation.

The patient was placed in a supine position and a lower midline incision from the umbilicus to symphysis pubis was performed. After opening the abdomen, a 6cm retroperitoneal mass was detected occupying the right presacral space and compressing the vascular structures. The ureter was identified and protected. The vascular structures were thoroughly dissected around the lesion. The mass was encapsulated with no invasion of the adjacent organs (Fig. 2). We performed a digital enucleation of the tumor through a capsulotomy.

**Fig. 3:**
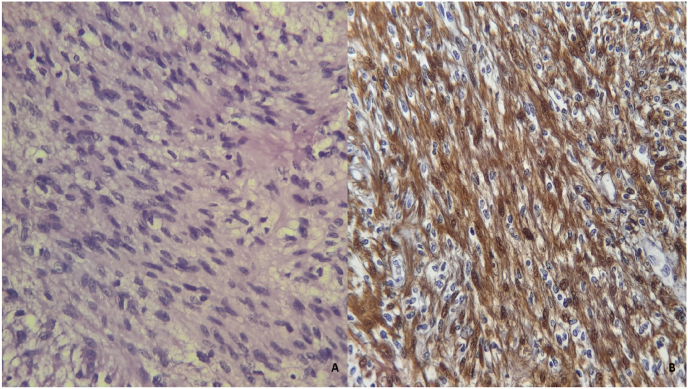
Schwann cells, fusiform with imprecise boundaries, with hyperchromatic nuclei (A) and Tumor cells express PS100 intensely and diffusely (B).

Pathology revealed a well-encapsulated tumor of interwoven spindle cell bundles. There were areas of calcification within the tumor. These features were typical of a degenerative schwannoma, and this was confirmed on immunohistochemistry with component cells staining strongly for S-100 protein. There were no features of malignancy (Fig. 3).

The post-operative period was uneventful. A follow-up examination at 6 months showed no signs of numbness or weakness in the right leg. The previously described pain totally regressed.

## 3 Discussion

Schwannoma is a rare nerve sheath tumor (NST), as it represents 0.3 to 0.4 cases per 100,000 persons per year, with most cases presenting around the fifth decade, which is similar to our patient [2,3].

What is interesting in our case is also the pelvic nature of the mass. Indeed, sacral localization of this tumor is quite rare as it represents less than 3% of all schwannomas [4].

This unique location and the fact that there is plenty of space to develop in is why presacral schwannoma is quite an insidious tumor. As it is pointed out in our case the patient began experiencing right radicular pain 2 years prior to the final diagnosis. This non-specific and very common symptom was treated as such with antalgics and non-steroidal anti-inflammatory. This is concurring with the literature, as some authors suggested that the onset of clinical manifestations ranges from 1 to 7 years according to literature with an extreme of 25 years in one case [5]. This is probably why incidental discovery is not uncommon for this particular tumor [6].

Now this delay in the diagnosis may be acceptable since schwannomas are mostly benign but bear in mind that 5–18% of them are associated with neurofibromatosis, which may become malignant [7].

The rareness of the disease and its pelvic location made it challenging for us to find conclusive recommendations to help with our management, but we did find Klimo et al. proposition for the classification of sacral NSTs interesting. Klimo et Al. suggested a classification scheme based on their location regarding the sacrum and were so divided into three types. Type III, as depicted in the classification, is primarily located in the presacral/retroperitoneal region similarly presented in our case [5]. Now this classification's aim in our understanding is to help physicians choose the right approach for this tumor. In our case, for a type III tumor, an anterior approach through laparotomy was necessary to thoroughly dissect all surrounding organs and major blood vessels thus preventing injuries and in operative complications.

This classification helped us in preoperative planning, involving a multidisciplinary approach with a neurosurgeon, an orthopedist, and a general surgeon. We did not need the intervention of a urologist in operative, but we did find it useful to put a double J stent in the right ureter prior to the operation to prevent its injury during the pelvic dissection.

What we find interesting in our case is that although authors suggested that sacrificing nerve roots carries a minimal risk of debilitating postoperative neurologic deficit [8], we chose to preserve them and went for a more conservative approach by doing an enucleation through a capsulotomy.

Now we are aware that this approach may expose to the risk of recurrence as it may be considered as a subtotal resection. This was noted in Conti P et l. Analysis that concluded to a 4.5% recurrence [9], all of which had subtotal resection. But we are however confident that the tumor was successfully removed and more important is the satisfaction of the patient for whom all symptoms disappeared without any functional impairment.

However, we do plan on conducting an annual surveillance regimen for our patient by clinical assessment and an MRI to detect an eventual local recurrence at an early stage of its evolution making reoperation easier.

## 4 Conclusion

Although schwannoma is a benign lesion, it may become malignant, especially when associated with neurofibromatosis making its surgical removal primordial. Its pelvic location may make its diagnosis delayed due to non-specific symptoms mainly through compression of local organs. Its surgical management can be challenging due to large size tumors with adherence to peritoneal and retroperitoneal organs. Quality of the resection is important in the recurrence and necessity for reoperation. A multidisciplinary approach is therefore recommended to ensure optimal treatment.

## Ethical approval

Not applicable.

## Sources of funding

No sources of funding.

## Authors contribution

Magherbi Houcine, CONCEPTUALISATION, REDACTION, DATA CURATION, PROJECT ADMINISTRATION.

Ouadi Yacine, CONCEPTUALISATION, REDACTION, DATA CURATION, PROJECT ADMINISTRATION.

Hammami Mahdi CONCEPTUALISATION, REDACTION.

Zehani Alia PHOTOGRAPHY RENDERING.

Boukriba Seif PHOTOGRAPHY RENDERING, DATA CURATION.

Zaraa Mourad DATA CURATION.

Fterich Fadhel Samir SUPERVISION, VALIDATION, VISUALISATION.

Montasser Kacem SUPERVISION, VALIDATION, VISUALISATION.

## Registration of research studies

1. Name of the registry:
2. Unique Identifying number or registration ID:
3. Hyperlink to your specific registration (must be publicly accessible and will be checked):

## Consent

Written informed consent was obtained from the patient for publication of this case report and any accompanying images. A copy of the written consent is available for review by the Editor-in-Chief of this journal on request.

## Guarantor

OUADI YACINE.

## Provenance and per review

Not commissioned, externally pee-reviewed.

## Declaration of competing interest

All authors declare that they have no any conflicts of interest.
